# Strong oviposition preference for *Bt* over non-Bt maize in *Spodoptera frugiperda* and its implications for the evolution of resistance

**DOI:** 10.1186/1741-7007-12-48

**Published:** 2014-06-16

**Authors:** Pilar Téllez-Rodríguez, Ben Raymond, Ivis Morán-Bertot, Lianet Rodríguez-Cabrera, Denis J Wright, Carlos G Borroto, Camilo Ayra-Pardo

**Affiliations:** 1 Centre for Genetic Engineering and Biotechnology (CIGB), Havana 10600, Cuba; 2 Division of Biology, Department of Life Sciences, Faculty of Natural Sciences, Imperial College London, Silwood Park campus, Ascot, Berkshire SL5 7PY, UK

**Keywords:** *Bacillus thuringiensis*, GM crops, High dose/refuge strategy, Oviposition behavior, Population dynamics, Resistance management, Transgenic crop

## Abstract

**Background:**

Transgenic crops expressing Bt toxins have substantial benefits for growers in terms of reduced synthetic insecticide inputs, area-wide pest management and yield. This valuable technology depends upon delaying the evolution of resistance. The ‘high dose/refuge strategy’, in which a refuge of non-Bt plants is planted in close proximity to the Bt crop, is the foundation of most existing resistance management. Most theoretical analyses of the high dose/refuge strategy assume random oviposition across refugia and Bt crops.

**Results:**

In this study we examined oviposition and survival of *Spodoptera frugiperda* across conventional and Bt maize and explored the impact of oviposition behavior on the evolution of resistance in simulation models. Over six growing seasons oviposition rates per plant were higher in Bt crops than in refugia. The Cry1F Bt maize variety retained largely undamaged leaves, and oviposition preference was correlated with the level of feeding damage in the refuge. In simulation models, damage-avoiding oviposition accelerated the evolution of resistance and either led to requirements for larger refugia or undermined resistance management altogether. Since larval densities affected oviposition preferences, pest population dynamics affected resistance evolution: larger refugia were weakly beneficial for resistance management if they increased pest population sizes and the concomitant degree of leaf damage.

**Conclusions:**

Damaged host plants have reduced attractiveness to many insect pests, and crops expressing *Bt* toxins are generally less damaged than conventional counterparts. Resistance management strategies should take account of this behavior, as it has the potential to undermine the effectiveness of existing practice, especially in the tropics where many pests are polyvoltinous. Efforts to bring down total pest population sizes and/or increase the attractiveness of damaged conventional plants will have substantial benefits for slowing the evolution of resistance.

## Background

Transgenic plants expressing the *Bacillus thuringiensis* (Bt) Cry toxins (Bt crops), first commercialized in 1996, represent a technological revolution in agricultural pest control, and can confer improved pest management, reduced pesticide applications, increased yields and area-wide reductions in pest populations [1-3]. By 2012, Bt crops were planted on about 70 million hectares worldwide [4]. Currently, the market of Bt crops is dominated by maize and cotton cultivars engineered to target Lepidoptera and/or Coleopteran pests. The primary threat to the ongoing success of Bt crops is the evolution of resistance by insect pests [5] and resistance management strategies are a legal requirement of the licensing of transgenic crops in many countries. Bt resistance is typically controlled by single large effect genes, with resistance being recessive at high doses of Cry toxin [6]. Regulatory agencies commonly demand that Bt cultivars express Cry toxins at a dose that will impose near completely recessive resistance, that is, be high enough to kill nearly all heterozygous resistant insects. In conjunction with these high doses, farmers are required to plant a refuge area of a non-Bt variety of the same crop in close proximity to the Bt crop [7]. Under this ‘high dose/refuge strategy’, the more abundant Bt susceptible adults emerging from the refuge will tend to mate with any resistant homozygous moths emerging from the Bt crop field [7,8]. The resulting heterozygous progeny should still be susceptible to the high dose of Bt toxins. So far, this strategy has successfully delayed resistance for Bt crops in many key target species [5,7].

However, field-evolved resistance to crops expressing Bt toxins has occurred at least in five cases where both failed insect control and an increased frequency of resistance genes have been recorded [9,10]. Notably, one of first documented cases of insect-resistance in Bt crops was in fall armyworm *Spodoptera frugiperda* (J.E. Smith) (Lepidoptera: Noctuidae) in Puerto Rico [11]. Resistance enabled *S. frugiperda* to survive and feed on Bt maize expressing Cry1F toxin in the field and led to the withdrawal of this product from Puerto Rico [11]. There are believed to be two main causes for resistance problems in general. First, growers may not strictly adhere to the requirements for planting refugia [12,13]. Second, toxin doses have been too low or variable to consistently kill heterozygous resistant insects [5,11,12].

Several oviposition preference studies have found adult females cannot discriminate between transgenic and near isogenic conventional cultivars [14-16]. However, these experiments only investigated the number of eggs on undamaged plants. This methodology, therefore, ignores any potential effects of subsequent larval feeding. Since many Bt crops kill newly hatched larvae soon after emergence, the feeding damage caused by larvae is likely to differ between conventional and Bt crops as the growing season progresses. This is significant as the volatiles released during larval feeding can deter oviposition in adult female Lepidoptera, a behavior that can reduce intra-specific competition or reduce detection by natural enemies that use plant volatiles as foraging cues [17,18].

In the present study, we tested the hypothesis that oviposition rates of *S. frugiperda* would respond to differences in damage levels incurred by Bt and non-Bt maize under field conditions. The field study covered both dry and wet growing seasons over four years in the Caribbean island of Cuba, which has similar growing conditions to neighboring Puerto Rico, where resistance to Cry1F maize was first documented [11]. *S. frugiperda* is the primary pest of maize (*Zea mays*) in the tropical region of the western hemisphere, where frequent insecticide applications are needed to maintain the population below economic thresholds [19]. We recorded oviposition rates, crop damage and larval numbers of *S. frugiperda* in Bt maize and its conventional refuge in young maize plants. We used these data to estimate in-field survivorship from egg to third instar and to inform simulation models that explored the impact of non-random damage-avoiding oviposition on the evolution of resistance under a high dose/refuge management strategy.

## Results

### Oviposition preference for Bt-maize in *S. frugiperda* correlates with the level of feeding damage in the refuge

**Figure 1:**
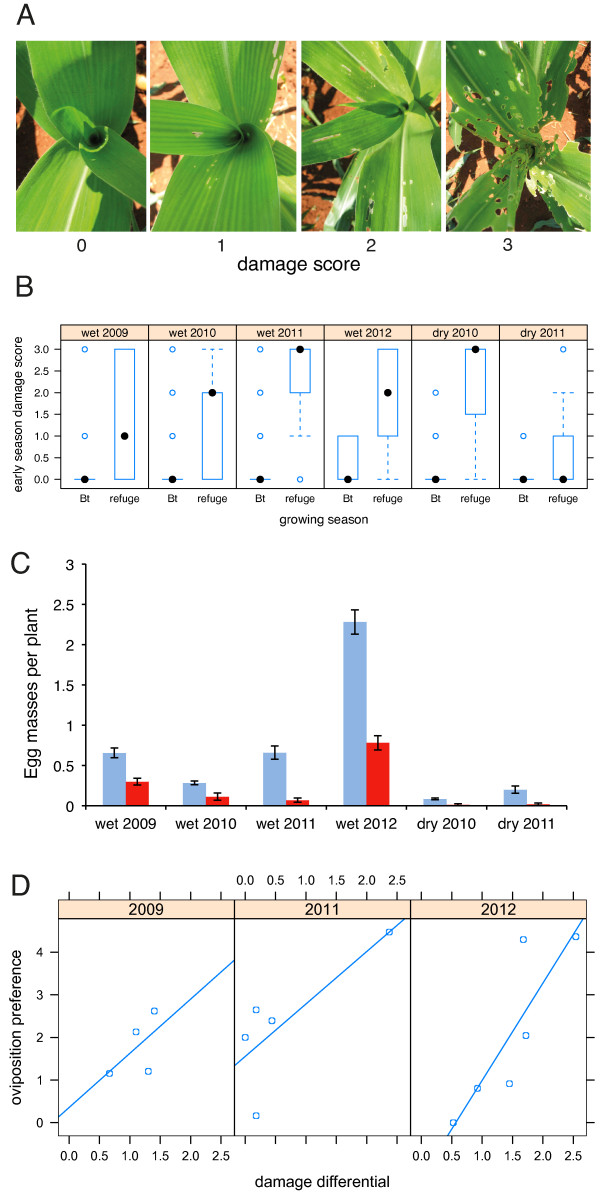
**Feeding damage and oviposition preference of *S. frugiperda *in transgenic Bt plants and conventional refuge plants. (A)** The damage scores of representative maize plants. **(B)** The variation in damage scores between transgenic Bt plants and conventional refugia plants. Box and whisker plots display medians (solid circles), lower and upper quartiles (boxes), the range (whiskers) and outliers (open circles). **(C)** Fall armyworm lay fewer egg masses on Bt plants (red bars) than on conventional plants (blue), than would be predicted by chance (Pearson’s χ^2^ = 92.9, *df* = 5, *P* < <0.0001), errors bars are ± SE. **(D)** The strength of preference for laying eggs in *Bt* maize increases with crop damage in the refuge within the growing season (overall slope = 1.4, SE = 0.38, *t* = 3.67, *P =* 0.0037). Data are from the wet growing seasons in 2009, 2011 and 2012. After taking the square roots of egg mass counts, preference is defined as egg masses per plant in *Bt* crop - egg masses per plant in refuge, while the damage differential is the difference in mean damage scores.

Damage to the vegetative crop was assessed on a four-point visual scale (Figure 1A). Data from the first observation each season (20 to 25 days post-planting) showed that transgenic FR-Bt1 plants were almost entirely undamaged, while the conventional variety FR-28 suffered a wider range of damage levels (Figure 1B). Statistical comparisons of damage scores were made twice per season using Kruskal Wallis non-parametric tests. Every comparison showed highly significant differences [see Additional file 1: Table S1], although damage scores were slightly reduced at the later observation period.

Scouting of egg masses allowed us to assess oviposition rates in Bt and the conventional parts of the crop. The number of egg masses per plant was significantly higher in Bt compared to non-Bt fields over six growing seasons (Figure 1C). Bias coefficients, which reflect the proportional reduction in egg counts in refugia relative to random expectation (see Methods), varied from 0.16 to 0.59, with a mean of 0.47. In other words, over the course of this study we recorded 145 egg masses in refugia, only 47% of the total of 309 masses predicted from a hypothesis of random oviposition. Crop damage levels varied within season, so we were also able to assess whether oviposition patterns responded to changing levels of damage in refugia. We analyzed data from the three seasons with the highest abundance of *S. frugiperda* and in which experimental plots were planted with 50% conventional refugia. The preference for Bt maize was significantly positively correlated with levels of damage in conventional refugia (Figure 1D, *P* = 0.0036, *df* = 1, likelihood ratio 8.49). This analysis also indicates that there were no intrinsic differences in preference for Bt maize over conventional maize, since when both crop types were undamaged oviposition rates were similar. This was statistically supported by our fitted model, in which the intercept was not significantly different from zero (value = 0.47, SE = 0.63, *t* = 0.75, *P* = 0.47) indicating random oviposition on undamaged conventional and Bt maize.

We inferred survivorship of *S. frugiperda* larvae from scouting observations and from the assumption that each egg mass contains, on average, 150 fertile eggs [20]. Survival on Bt maize ranged from 0.006 to 0.0003 [see Additional file 1: Table S2]. Moreover, comparisons between years showed that natural mortality in the conventional crop was strongly negatively density-dependent, with mortality following an inverse power law [see Additional file 2: Figure S1].

### Biased oviposition has substantial effects on the evolution of resistance

We investigated the effects of damage-avoiding oviposition on the evolution of resistance using computer simulation models. These models were informed by our field data and by previous reports of the genetics of Bt resistance in *S. frugiperda*[11,21]. Larval population densities in conventional refugia determined oviposition bias in these models. We considered how different pest management regimes with different forms of density dependence would affect the evolution of resistance, as these will impact larval densities, leaf damage and damage-avoiding oviposition behavior. We considered three scenarios: a spray threshold model in which application of synthetic pesticides in refugia was based on the number of egg masses laid per crop plant; a natural predation model with density-dependent mortality of larvae in refugia only (based upon the power law inferred from field data); and a predation model in which there was density-dependent mortality occurring throughout the *Bt* crop and the refuge in addition to the mortality imposed by the plant incorporated Bt toxin.

**Figure 2:**
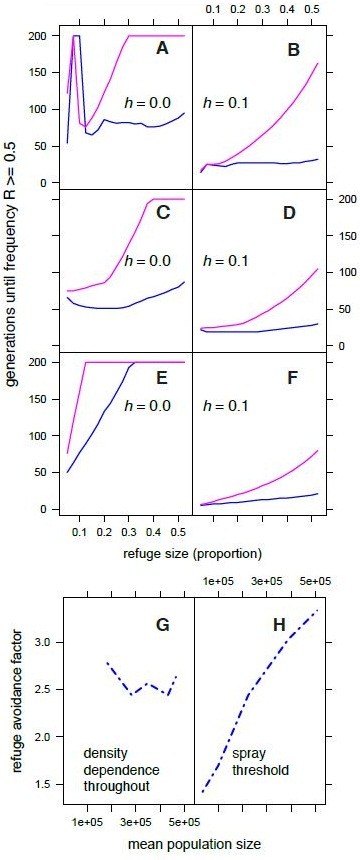
**Evolution of resistance to a single *Bt *toxin crop under random oviposition (magenta lines) and damage-avoiding oviposition (blue lines).** Models simulated three scenarios for pest management with different forms of density dependence under fully recessive (*h* = 0) or partially dominant resistance (*h =* 0.1). In **A** and **B** pest management in refugia uses a density-dependent spray threshold of 0.25 egg masses per plant; in **C** and **D** natural enemies cause density-dependent mortality in refugia while in **E** and **F** natural enemies cause density-dependent mortality throughout the refugia and the *Bt* crop. The response variable is the number of generations required for the resistance gene frequency (R) to equal or exceed 0.5, up to maximum of 200. Note that a value of 200 is returned if the frequency of resistance never exceeded 0.5. Fecundity was set at 300 eggs per female and the initial resistance gene frequency was 0.001. **G** and **H** show the relationship between population density and avoidance of refugia under the two modeling scenarios with *h =* 0.1. Parameter values in the last two panels are calculated before the evolution of full resistance, that is, from generation 1 until the time point at which the frequency R > = 0.5. The refuge avoidance factor is *1/b* and represents the multiplicative reduction in eggs laid in the refuge relative to random expectation, so that 1 indicates random oviposition and 3 indicates a three-fold reduction in egg counts relative to random expectation.

When oviposition was random, we generally recovered the typical result in which increasing refuge size provided better control of resistance (Figure 2). The one exception to this rule was that the evolution of fully recessive resistance was prevented at very low refuge sizes under the spray threshold scenario (Figure 2A). This occurred because simulated pests formed ‘population sinks’ with negative population growth at very low refuge sizes so that population sizes were never large enough (<10,000 adults) to produce any resistant homozygotes. Refugia of 10% to 30% of the total crop area were typically required to control the evolution of resistance when initial resistance was fully recessive (Figure 2A,C,E), although stable management of resistance was not possible under partially dominant resistance (Figure 2B,D,F). Damaging-avoiding oviposition accelerated resistance evolution, and undermined resistance management. Only under the most favorable conditions (recessive resistance, refugia >30% and density dependence throughout the crop) was stable resistance management possible with damage-avoiding oviposition (Figure 2E). When there was density dependence throughout the crop refuge size and the dominance of resistance appeared to interact: refuge size was a more important parameter as dominance decreased [see Additional file 3: Figure S2].Moreover, the problems caused by damage-avoiding oviposition could not typically be resolved by planting larger refugia, especially when resistance was partially dominant. This insensitivity to refuge size occurred because increasing refuge sizes increased adult population density and overall pest pressure. Higher population densities led to increased damage rates and more biased oviposition. With fully recessive resistance increasing refugia size had some benefits, especially when we imposed some density-dependent mortality throughout the crop (Figure 2C,E). The natural predation scenario could provide better resistance management at higher refuge size because strong density-dependent mortality ensured that oviposition bias did not increase with population size (Figure 2G). Conversely, under a spray threshold regime, the proportion of eggs laid in refugia decreased dramatically with population size, thereby offsetting the benefits of planting a larger refuge (Figure 2H). Note that under both scenarios egg counts in Bt plants were at most three times greater than expected by chance, a fairly conservative maximum given our field data.

### Pest population dynamics strongly influence resistance evolution

**Figure 3:**
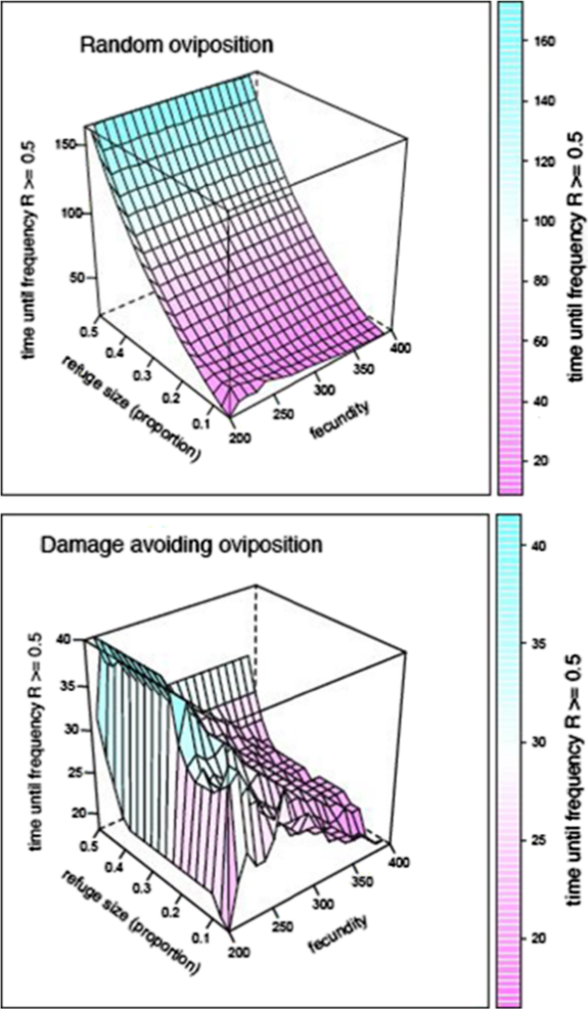
**The effects of random and damage-avoiding oviposition behavior and female fecundity on evolution of resistance under the sprayed refuge scenario.** In these simulations the spray action threshold for refugia was 0.25 egg masses per plant, the initial frequency of resistance = 0.001 and we assumed partially dominant resistance (*h* = 0.1).

We further explored the impact of population dynamics on the evolution of resistance in a series of sensitivity analyses. With random oviposition under the spray-threshold scenario, female fecundity affected evolution of resistance when it led to negative population growth under partially dominant (Figure 3) and fully recessive resistance [see Additional file 4: Figure S3]. However, under damage-avoiding oviposition, increased fecundity reduced the time to evolve resistance over a range of parameter values. Simulation results were, however, relatively insensitive to spray thresholds, so that decisions to minimize damage by spraying lightly infested refugia are not predicted to improve resistance management [see Additional file 5: Figure S4]. Spray thresholds were only important with large refugia under damage-avoiding oviposition; under those conditions higher spray thresholds (>0.6 egg masses per plant) tended to delay resistance [see Additional file 5: Figure S4].

## Discussion

Here, we have shown that *S. frugiperda* has a strong oviposition preference for undamaged *Bt* maize over damaged conventional maize planted as a refuge for resistance management. Oviposition by *S. frugiperda* affects the production of plant volatiles in maize [22]. Moreover, ovipositing females show a preference for the volatiles of undamaged maize plants [23]. Early reports have also described how the egg-laying behavior of this species changes when larval densities are high [24]. We controlled for the genetic background of the *Bt* and conventional crop species, so that differences in maize variety other than the presence or absence of Cry1F are unlikely to have affected insect behavior. Since oviposition preference within season also increased with the level of leaf damage in the conventional refuge the most plausible explanation for increased egg laying in the Bt crop field is that females are avoiding plants previously damaged by conspecifics. This behavior makes good adaptive sense for ovipositing females, as larval progeny on damaged plants may suffer increased competition for food and more attention from foraging parasitoids [25,26]. One caveat of our observations is that it is uncertain whether differences in oviposition rates will also hold true when there are much greater distances between refugia and Bt crops. The ‘in-field’ refugia that were used in this study are relatively rare in some maize cropping areas [27]. That being said, all refugia must be planted close enough to Bt crops in order to facilitate random mating between adults emerging from transgenic and conventional plants, so that females on the wing should be able to readily sample volatiles from both crop types.

Modeling of the evolution of resistance to Bt under damage-avoiding oviposition has previously assumed that adults will prefer to lay eggs on conventional rather than Bt crops [28], perhaps because Bt and its toxins have been shown to deter larval feeding [29,30]. This earlier theoretical work is consistent with our results, in that resistance management improves as more eggs are laid in refugia [28]. However, this earlier hypothesis of avoidance of Bt crops is unsupported [14-16]. Damage on Bt crops is typically less than on their non-Bt counterparts. Given the many reports of avoidance of damaged plants or damage-induced volatiles by adult Lepidoptera [17,18,26,31-34], the opposite prediction is more likely to hold: reduced leaf damage in Bt crops will attract increased rates of oviposition. Since leaf damage is determined by larval density, our modeling showed that the population dynamics of pest species in conventional refugia have a strong impact on the efficacy of the high dose/refuge management strategy.

This conclusion is supported by the details of the evolution of resistance to Bt maize by *S. frugiperda* in Puerto Rico [11]. In that case, population densities of *S. frugiperda* were high prior to reports of control failure [11]. This population outbreak was compounded by a drought, which eliminated natural refugia in wild grasses, concentrating larval populations onto irrigated maize crops. This would have led to very high damage levels on conventional maize plants and a substantial weakening of the efficacy of any planted refugia. In Puerto Rico, the evolution of resistance occurred within 30 to 40 generations [11]. Simulation models that incorporate damage avoidance predict the evolution of resistance within a similar period of time (27 generations), when we make assumptions that fit with the Puerto Rican experience, that is, partial dominance and a sprayed 20% to 30% refuge (N. Storer, personal communication). In contrast, if we assume random oviposition, the predicted time range for the evolution of resistance is 39 to 65 generations, a range which is largely outside the Puerto Rican estimate. This correspondence between field data and modeling is reassuring and suggests damage avoidance could have accelerated the evolution of resistance to Cry1F in Puerto Rico. However, this match must be treated with caution as it was not our intention to explicitly simulate the Puerto Rican situation, and important parameters, such as the initial frequencies of resistance in Puerto Rico, were unknown.

Our results have several implications for resistance management in Bt crops. One clear recommendation is that there are substantial gains to be made by improving area-wide pest population control, as lower population sizes will decrease the difference in damage levels between Bt crops and refugia. A second implication is that resistance management strategies that are less dependent on refugia will be less vulnerable to non-random oviposition. One such possible strategy is the large-scale release of transgenic female-specific RIDL (release of insects with dominant lethality) insects for population control, as mating with large numbers of Bt susceptible released males is the mechanism that lowers the frequency of resistance in the field [35]. Insect population dynamics and density dependence were important for resistance evolution, but had the most impact when we assumed recessive resistance. Large, unsprayed conventional refugia with control of pests by natural enemies could provide good resistance management under damage-avoiding oviposition, if we assumed there was also some density-dependent mortality in the *Bt* portion of the crop. One caveat here is that our endemic population of *S. frugiperda* was relatively well controlled by density-dependent larval mortality. This natural regulation can be disrupted by the over-use of broad-spectrum insecticides or by the migration of *S. frugiperda*, since migrating populations can escape natural enemy control [24]. Nevertheless, when natural enemy regulation is poor, high spraying rates in refugia did not benefit resistance management in our simulations.

The presented simulation results depend upon the naïve assumption that pest population dynamics are entirely driven by population processes in crops, an assumption that will be true to a greater or lesser extent in different pest species and at different times. For example, while *S. frugiperda* can feed on a range of wild grass species in the wet season [24], during the dry season this species is more dependent on irrigated crops [11]. Pests with a narrow host range are clearly more dependent on agro-ecosystems; however, agricultural practice can have substantial impacts on the populations of polyphagous pests [1,2]. In the event that agricultural practice has no impact on pest population dynamics, it would still be beneficial to plant the largest non-Bt refuge possible, as larger refugia would not affect oviposition bias.

While we found that damage-avoiding oviposition has the potential to undermine current resistance management strategies, there are several possibilities that might mitigate this effect. Maize plants, for example, show considerable genetic variation in the extent and make-up of volatile release after damage [36]. It may, therefore, be possible to plant non-Bt refugia with crop plants that remain attractive to ovipositing females after being damaged by larvae. Refugia are more effective when plants are less damaged and insect populations reduced. Efforts to bring down regional population sizes in an integrated pest management program (IPM) be they via pheromone trapping, habitat management (control of alternative hosts) or mechanical ‘pupae busting’ , will all yield benefits for diverse insect pests in the face of damage averse oviposition [37]. We expected that lowering the density of insects in refugia by imposing tighter spray thresholds might also mitigate the impact of damage-avoiding oviposition. However, simulation results were largely insensitive to refugia spray thresholds, possibly because increasing spray rates also decrease insect population sizes in refugia [see Additional file 3: Figure S2] or because density-dependent mortality in refugia can itself accelerate the evolution of resistance [38,39].

## Conclusions

Modern multiple toxin Bt crop varieties are expected to improve resistance management [40,41]. Nevertheless, these crops are not expected to be immune to the evolution of resistance [10,42] and non-Bt refugia are still required for effective resistance management [40,41]. In both single and multiple toxins systems the efficacy of any planted refuge will be reduced by oviposition bias, especially where pests are polyvoltinous or have overlapping generations and are, therefore, capable of responding to the damage caused by larvae feeding earlier in the season. A number of insect species are deterred from laying eggs on plants damaged by conspecific larvae [17,18,26,31-34]. These species include prominent target pests for a wide range of Bt crops, such as *Heliothis virescens, S. frugiperda, Spodoptera littoralis* and *Ostrinia nubilalis*[17,18,23,34]. For *H. virescens* and *O. nubilalis* current resistance management practices for slowing the evolution of resistance to Bt crops appear to be effective [1,43], despite possible issues with damage-avoiding oviposition. This could be because area-wide management of these pests has been effective enough to prevent high levels of damage in refugia [1,2], because the results here which are based on a small-scale field trial may not scale up to larger refugia or because bivoltine dynamics limit the opportunities for damage-avoiding oviposition to a single generation per year [44]. Despite these caveats it is likely that the impact of oviposition behavior on the evolution of resistance to Bt crops has been underestimated and needs careful re-evaluation.

## Methods

### Plants

The Bt (Cry1F) maize variety FR-Bt1 (referred to as Bt maize) along with the Cuban open-pollinated variety FR-28 (referred to as non-Bt maize) [45] were used in all the experiments. FR-Bt1 was produced in the CIGB by crossing plants of a commercial Bt maize bearing the TC1507 event into FR-28 plants and back-crossing four additional times into this conventional maize variety. Before each backcross, FR-Bt1 was selected for Bt Cry1F expression by ELISA immunoassay (Agdia Inc., Elkhart, IN, USA). Based on the number of backcrosses, the resultant FR-Bt1 plants contain 96.75% of the genetic material of the FR-28 genotype.

### Study area and experimental conditions

The experiments were conducted at the CIGB experimental field station from 2009 to 2012. Bt and non-Bt maize cultivars were planted during the second week of January (for the dry season) and first week of May (for the wet season). Plants were arranged in a randomized complete block design with four replicates. Each field plot had three rows, and row size ranged from 4 to 10 m long. The distance between plots of Bt corn and non-Bt corn was five meters. All studies followed standard agronomic practices for maize. Seeds used for testing were treated only with a commercial fungicide (Poncho 250, Bayer CropSciences, Monheim, Germany) and no insecticides were applied to any part of the plots. Natural rainfall was supplemented by irrigating weekly with 7 cm of water as needed, particularly during the dry season.

The number of egg masses, the rank of leaf-feeding injury and the larval development stage were scored from the middle row of the four independent plots repeatedly over the growing season. Observations began at the V_3_ (three true-leaves) stage and were taken weekly until no new egg masses were found for two consecutive weeks. To prevent duplicate counting, each new egg mass was circled on the leaf with a waterproof marker. The total plants sampled in the Bt crop and conventional refuge respectively were: wet season 2009, N *=* 376 and N *=* 236; dry season 2009, N *=* 175 and N *=* 170; wet season 2010, N *=* 670 and N *=* 80; dry season 2010, N *=* 530 and N *=* 53; for all remaining seasons N *=* 100 in both sections of the crop. The damage from *S. frugiperda* herbivory was assessed at around 20 days after planting and on the last day any significant damage was detected. Leaf damage was estimated using a four-point damage visual-rating scale (1 to 4), where a score of 1 indicates no visible damage, while a score of 4 represents >90% destruction of whorl and furl leaves (Figure 1A). For the estimates of larval survival, a mean of 150 eggs per egg mass was assumed. Larval scouting began at the V3 stage and was maintained until one week before initiation of flowering. Instars were determined by measuring larval size and head capsule width [46]. The larvae were considered dead if they did not move when touched with a needle.

### Data analysis

Data on oviposition rates were analyzed using Pearson’s Chi-squared test, using mean egg masses per plant in the whole crop to calculate expected frequencies. All statistical tests were carried out in R v 2.6.2 [47]. Mixed models of oviposition preference within season used mean damage in the refuge as a fixed factor and year of planting as a random effect. Models with different fixed effect structures were compared using likelihood ratio tests following maximum likelihood model fitting. Final model fitting and model assumptions were checked with graphical analyses [48].

### Simulation models

The deterministic two-patch population models followed a published framework [49] and were carried out in R v 2.6.2. A single gene determined resistance. Storer *et al.* (2010) estimated the dominance of resistance in terms of survival on Cry1F plants as between 0.07 and 0.14 [11]. All models considered at least two values for dominance: completely recessive (*h* = 0) and partially dominant (*h* = 0.1). Further sensitivity analyses on the effects of dominance were conducted as appropriate [see Additional file 3: Figure S2]. Fitness costs of resistance were recessive and fixed at a 25% reduction in larval survival. We assumed random mating and used data from previous reports of CryIF resistance [11,21] and our field observations to determine life history parameters [see Additional file 1: Table S3]. Pest populations in the model were closed to immigration and emigration. We assumed non-overlapping generations and imposed a minimum population size of 4,000 adults and a carrying capacity of 400,000 adults at the end of each generation.

The oviposition bias coefficient, *b,* was calculated as 1 - (number of adults in refuge/number of plants in refuge) and was bounded by 0.2 and 1.0, after carrying capacity had been imposed on adult populations. The proportion of total oviposition in refugia was calculated according to *b X p* where *p* is the proportional size of the refuge. Oviposition in the Bt crop was calculated as 1 - (b X p). Thus, a bias coefficient of 1.0 was equivalent to random oviposition, while at a bias of 0.2, oviposition in refugia was reduced five-fold relative to random expectation. Field observations showed that oviposition rates in the summer growing seasons were reduced by two- to five-fold relative to random expectation.

We simulated a range of management scenarios that imposed different forms of density-dependent mortality on insect larvae. Density independent mortality followed the constant survival of 14% [see Additional file 1: Table S2]. Spray action thresholds for control of pests in refugia were calculated according to the number of egg masses per plant, assuming each female laid two egg masses of 150 eggs with a maximum of one egg mass per plant. These were informed by agriculture extension service recommendations for *S. frugiperda* across North America on the proportion of infested field corn plants (typically 25% to 75% of plants with damage) [50-52]. There was clear evidence for density-dependent larval mortality in experimental refugia from field observations of mortality from egg to third instar [see Additional file 2: Figure S1]. Mortality imposed by natural enemies in the refugia was based on a power law fitted to these data. We halved the overall survivorship relative to field conditions to account for the death of later instars, pupae and adults so that survival = 0.01*x*^-1.274^ where x is the total density of adults per plant in the whole crop, calculated after the imposition of carrying capacity. The field data could not reliably indicate whether overall mortality in the *Bt* crop was entirely density independent or incorporated a density-dependent component. We, therefore, also simulated a scenario in which density-dependent mortality occurred throughout the crop, with the survival of susceptibles calculated as = 0.00025*x*^-1.274^ [see Additional file 2: Figure S1] on the Bt portion of the crop. After the imposition of fitness costs, the mortality of resistant larvae was unaffected by toxin, as indicated by previous work [21].

## Competing interests

The authors declare that they have no competing interests.

## Authors’ contributions

PTR obtained the Bt-maize variety, prepared the documentation for the biosafety license to grow a Bt-maize in open field conditions, participated in the design of the study and performed the field experiments. BR analyzed the field data, conducted the simulation modeling and wrote the paper. IMB and LRC participated in obtaining the Bt-maize variety, and the biosafety license for experiments with Bt-maize in open field conditions and assisted with the field experiment. CGB participated in the study design and coordination of the field experiments. DJW participated in the study design and helped to draft the manuscript. CAP conceived and designed the study, and drafted the manuscript. All authors read and approved the final manuscript.

## Supplementary Material

Additional file 1: Table S1 Comparisons of crop damage; data are means from a four-point damage scale (0- no damage, 4- heavily damaged). Kruskal Wallis tests were conducted for data from no more than two time points per season: at around 20 days after planting and also on the last day any significant damage was detected. Key: **** *P* <0.0001, *** *P* <0.001. **Table S2.** Estimated survivorship from egg to third instar larva based on field observations over four independent plantings of Bt maize and conventional refuge. Survivorship is based on females laying an average of 150 eggs per egg mass. **Table S3.** Summary of simulation model parameters. Note: survival is given before effects of fitness costs are calculated. An oviposition bias of 1 is equivalent to random egg-laying behavior. Click here for file

Additional file 2: Figure S1 (A) Density-dependent survivorship of fall armyworm in the conventional maize refuge. Estimates of survivorship (diamonds) are based on scouting of larvae and on the assumption that each egg mass contains 150 eggs. (B) Estimates of larval survival in the experimental *Bt* crop (asterisks) showing the fitted density-independent and density-dependent mortality used in simulations. Click here for file

Additional file 3: Figure S2 The impact of dominance of resistance on the evolution of resistance in computer simulations when there is density-dependent mortality throughout the refuge and the Bt crop. (A) Under random oviposition increasing refuge size and decreasing dominance slow the evolution of resistance. (B) Under damage-avoiding oviposition these factors appear to interact more: increasing refuge size had a greater impact on delaying resistance as the dominance of resistance decreased. Click here for file

Additional file 4: Figure S3 The impact of population dynamics and biased oviposition on the evolution of resistance in computer simulations when resistance to Bt toxins is fully recessive. Here, we explored the relationship between female fecundity and refuge size under random (A) and damage-avoiding oviposition (B). As in previous models, simulation artefacts meant that the evolution of resistance was prevented in sink populations with small refugia. In general, larger refugia did not effectively prevent evolution of resistance when females were avoiding damaged plants. Simulation results were largely insensitive to female fecundity under random oviposition, while female fecundity was an important parameter under damage-avoiding oviposition. The spray action threshold was set at 0.25 egg masses per plant in all simulations. Click here for file

Additional file 5: Figure S4 The impact of population dynamics and biased oviposition on the evolution of resistance in computer simulations when resistance to Bt toxins is under partially dominant (*h* = 0.1, A, B) and fully recessive (*h* = 0) resistance (C,D). Here, we explored the relationship between the density dependent action threshold for spraying refugia (in egg masses per plant) and refuge size under random and damage-avoiding oviposition . As in previous models, larger refugia had a limited impact on the evolution of resistance when females were avoiding damaged plants. This result was relatively insensitive to spray thresholds, except when refugia were very large. Note that sprays are rarely applied with spray thresholds of 0.8 or above. Once again, small refugia could lead to the simulated populations becoming sinks, which also prevented the evolution of fully recessive resistance. Fecundity was set at 300 eggs per female in all models. Click here for file
