# Supplementary material for: Strong oviposition preference for Bt over non-Bt maize in Spodoptera frugiperda and its implications for the evolution of resistance
Source: BMC Biol. 2014 Jun 16;12:48. doi: 10.1186/1741-7007-12-48 (PMC4094916; doi:10.1186/1741-7007-12-48)
Supplement: Additional file 1: Table S1 — Comparisons of crop damage; data are means from a four-point damage scale (0- no damage, 4- heavily damaged). Kruskal Wallis tests were conducted for data from no more than two time points per season: at around 20 days after planting and also on the last day any significant damage was detected. Key: **** P <0.0001, *** P <0.001. Table S2. Estimated survivorship from egg to third instar larva based on field observations over four independent plantings of Bt maize and conventional refuge. Survivorship is based on females laying an average of 150 eggs per egg mass. Table S3. Summary of simulation model parameters. Note: survival is given before effects of fitness costs are calculated. An oviposition bias of 1 is equivalent to random egg-laying behavior. [file 1741-7007-12-48-S1.doc]

**Table S1**. Comparisons of crop damage, data are means from a four-point damage scale (0- no damage, 4- heavily damaged). Kruskal Wallis tests were conducted for data from no more than two time points per season: at around 20 days after planting and also on last day any significant damage was detected. Key: **** *P* < 0.0001, *** *P* < 0.001

| Season | Time after planting / days | Mean (se) damage score in *Bt* crop | Mean (se) damage score in refuge | χ^2^ (with 1 *df*) |
| --- | --- | --- | --- | --- |
| Winter 2009 | 24 | 0.01 (0.0002) | 0.58 (0.12) | 87.6 **** |
|  | 45 | 0 (0) | 0.78 (0.20) | 66.3 **** |
| Summer 2009 | 26 | 0.11 (0.015) | 1.40 (0.32) | 122.1 **** |
|  | 40 | 0.011 (0.002) | 0.66 (0.15) | 51.8 **** |
| Winter 2010 | 23 | 0.26 (0.05) | 2.15 (1.56) | 37.9 **** |
|  | 51 | 0.05 (0.008) | 0.99 (0.40) | 58.2 **** |
| Summer 2010 | 25 | 0.12 (0.008) | 1.38 (0.47) | 169.0 **** |
|  | 39 | 0.036 (0.004) | 0.70 (0.33) | 74.4 **** |
| Winter 2011 | 20 | 0.06 (0.006) | 0.39 (0.068) | 20.15 **** |
|  | 55 | 0 (0) | 0.31 (0.09) | 11.58 *** |
| Summer 2011 | 23 | 0.14 (0.03) | 2.37 (0.69) | 139.4 **** |
| Summer 2012 | 26 | 1.05 (0.24) | 2.54 (0.80) | 96.1 **** |
|  | 47 | 0.06 (0.01) | 1.45 (0.45) | 90.7 **** |

**Table S2.** Estimated survivorship from egg to third instar larva based on field observations over four independent plantings of Bt maize and conventional refuge. Survivorship is based on females laying an average of 150 eggs per egg mass.

|  | *Bt* crop survival (N egg masses) | Refuge survival (N egg masses) | Proportional planted as refuge (N plants sampled) |
| --- | --- | --- | --- |
| Winter 2010 | 0.006 (58) | 0.52 (1) | 0.1 (760) |
| Summer 2010 | 0.0009 (151) | 0.08 (6) | 0.1 (563) |
| Winter 2011 | 0.0003 (20) | 0.32 (2) | 0.5 (200) |
| Summer 2011 | 0.0009 (66) | 0.1 (7) | 0.5 (200) |
| Summer 2012 | 0.001 (228) | 0.013 (78) | 0.5 (200) |
| Mean | 0.0008 |  |  |

**Table S3.** Summary of simulation model parameters. Note survival is given before effects of fitness costs are calculated. An oviposition bias of 1 is equivalent to random egg-laying behaviour.

| Notation | Description | Values/Range |
| --- | --- | --- |
|  | *Population & genetic variables* |  |
| *S_s_* | Survival of SS insects exposed to *Bt* | 0.0008 |
| *S_r_* | Survival of RR insects exposed to *Bt* | 0.14 |
| *c_r_* | Multiplicative fitness cost of resistance | 0.75 |
| *h* | Dominance of resitance (survival) | 0-0.1 |
| *h_c_* | Dominance of fitness cost *c_r_* | 0 |
|  | Proportional size of conventional refuge | 0.05-0.5 |
| *R* | Initial resistance gene frequency | 0.001-0.002 |
|  | Fecundity | 2-400 eggs per female |
|  | Refuge spray threshold | 1.5-2.5 x10^5^ egg masses0.140 |
| *b* | Oviposition bias | 0.2-1.0 |
|  |  |  |
|  | *Population constants* |  |
| *K* | Maximum population size | 4x10^5^ adults |
|  | Minimum population size | 4x10^3^ adults |
|  | Initial population size | 1x10^4^ adults |
|  | Number crop plants | 1x10^6^ |
|  | Survival in sprayed refuge | 0.05 |
|  | Survival in refuge without sprays  or natural enemies | 0.14 |
