# Supplementary material for: Strong oviposition preference for Bt over non-Bt maize in Spodoptera frugiperda and its implications for the evolution of resistance
Source: BMC Biol. 2014 Jun 16;12:48. doi: 10.1186/1741-7007-12-48 (PMC4094916; doi:10.1186/1741-7007-12-48)

**Figure S1. (A)** Density dependent survivorship of fall armyworm in the conventional maize refuge. Estimates of survivorship (diamonds) are based on scouting of larvae and on the assumption that each egg mass contains 150 eggs. **(B)** Estimates of larval survival in the experimental *Bt* crop (asterisks) showing the fitted density independent and density dependent mortality used in simulations.

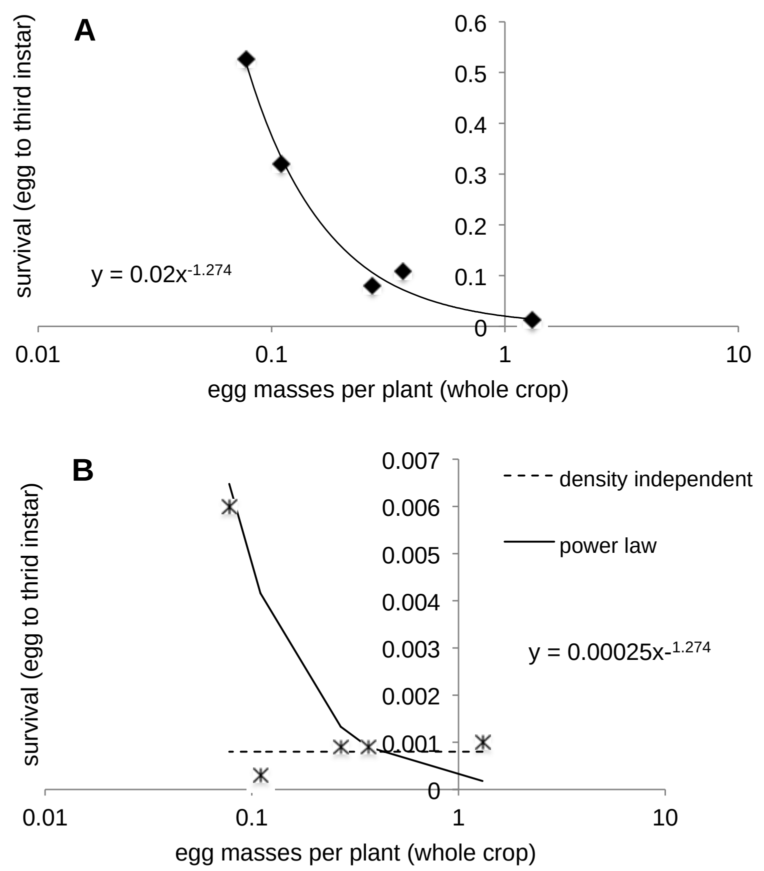

Supplement: Additional file 2: Figure S1 — (A) Density-dependent survivorship of fall armyworm in the conventional maize refuge. Estimates of survivorship (diamonds) are based on scouting of larvae and on the assumption that each egg mass contains 150 eggs. (B) Estimates of larval survival in the experimental Bt crop (asterisks) showing the fitted density-independent and density-dependent mortality used in simulations. [file 1741-7007-12-48-S2.pdf]
