# Supplementary material for: Strong oviposition preference for Bt over non-Bt maize in Spodoptera frugiperda and its implications for the evolution of resistance
Source: BMC Biol. 2014 Jun 16;12:48. doi: 10.1186/1741-7007-12-48 (PMC4094916; doi:10.1186/1741-7007-12-48)

Figure S2.   The impact of dominance of resistance on the evolution of resistance in computer simulations when there is density dependent mortality throughout the refuge and the Bt crop **(A)** Under random oviposition increasing refuge size and decreasing dominance slow the evolution of resistance (**B)** Under damage avoiding oviposition these factors appear to interact more: increasing refuge size had a greater impact on delaying resistance as the dominance of resistance decreased.

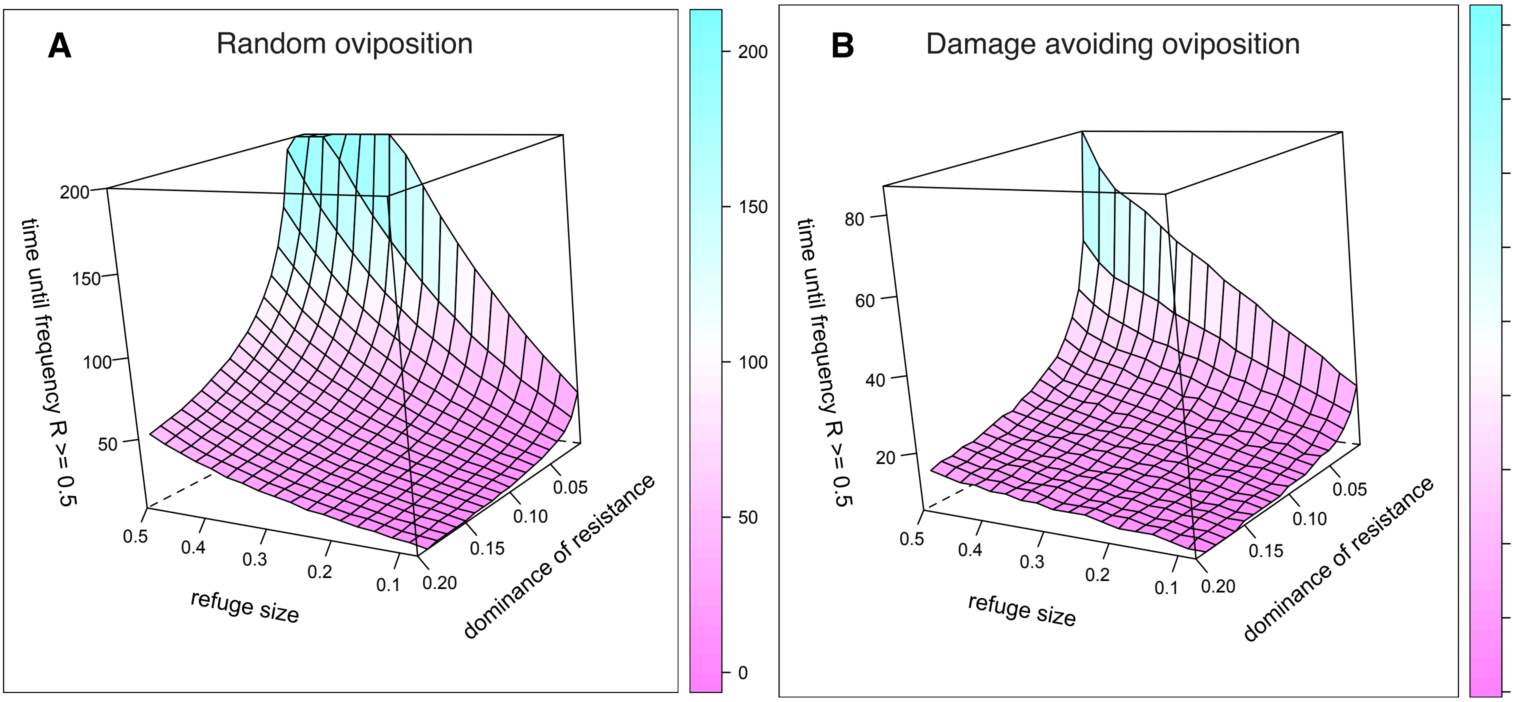

Supplement: Additional file 3: Figure S2 — The impact of dominance of resistance on the evolution of resistance in computer simulations when there is density-dependent mortality throughout the refuge and the Bt crop. (A) Under random oviposition increasing refuge size and decreasing dominance slow the evolution of resistance. (B) Under damage-avoiding oviposition these factors appear to interact more: increasing refuge size had a greater impact on delaying resistance as the dominance of resistance decreased. [file 1741-7007-12-48-S3.pdf]
