# Supplementary material for: Strong oviposition preference for Bt over non-Bt maize in Spodoptera frugiperda and its implications for the evolution of resistance
Source: BMC Biol. 2014 Jun 16;12:48. doi: 10.1186/1741-7007-12-48 (PMC4094916; doi:10.1186/1741-7007-12-48)

Figure S3. The impact of population dynamics and biased oviposition on the evolution of resistance in computer simulations when resistance to Bt toxins is fully recessive. Here we explored the relationship between the female fecundity and refuge size under random (**A**) and damage avoiding oviposition (**B**). As in previous models simulaton artefacts meant that the evolution of resistance was prevented in sink populations with small refugia. In general larger refugia did not effectively prevent evolution of resistance when females were avoiding damaged plants. Simulation results were largely insensitive to female fecundity under random oviposition, while female fecundity was an important parameter under damage avoiding oviposition. The spray action threshold was set at 0.25 egg masses per plant in all simulations.

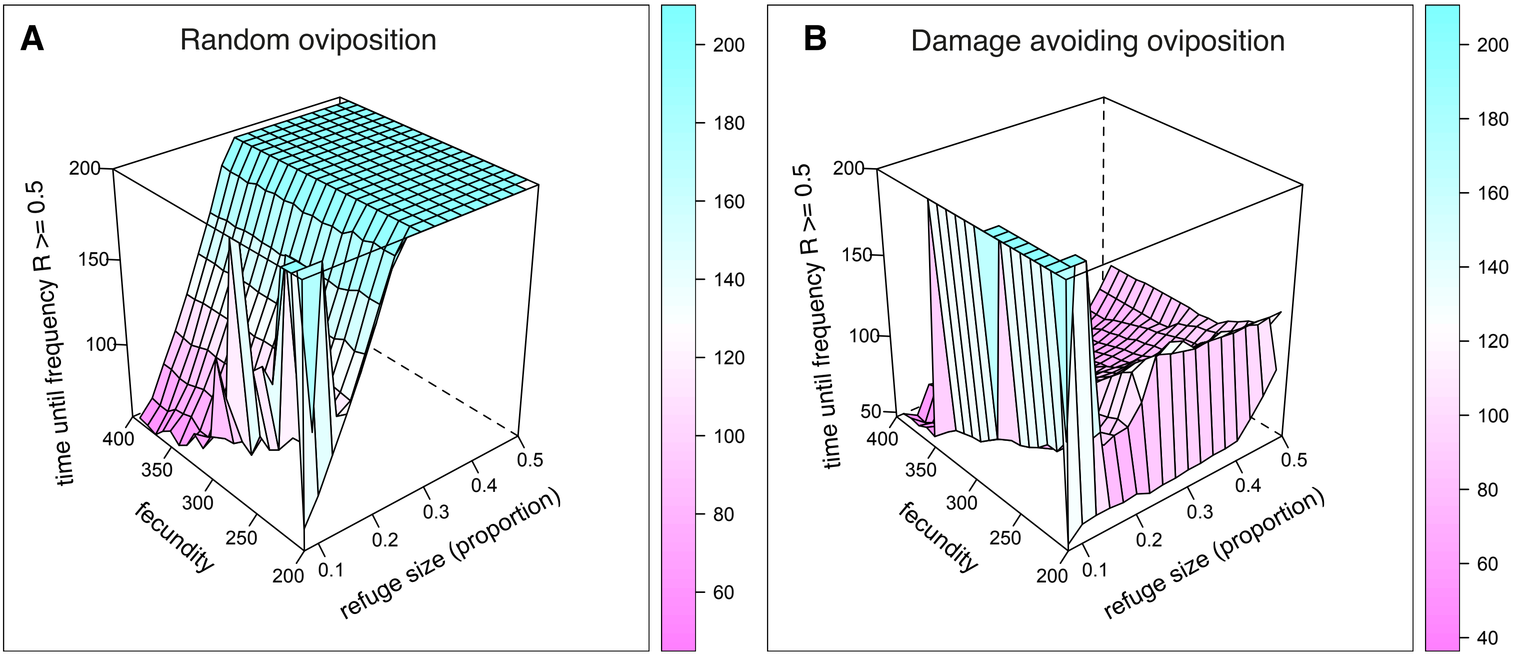

Supplement: Additional file 4: Figure S3 — The impact of population dynamics and biased oviposition on the evolution of resistance in computer simulations when resistance to Bt toxins is fully recessive. Here, we explored the relationship between female fecundity and refuge size under random (A) and damage-avoiding oviposition (B). As in previous models, simulation artefacts meant that the evolution of resistance was prevented in sink populations with small refugia. In general, larger refugia did not effectively prevent evolution of resistance when females were avoiding damaged plants. Simulation results were largely insensitive to female fecundity under random oviposition, while female fecundity was an important parameter under damage-avoiding oviposition. The spray action threshold was set at 0.25 egg masses per plant in all simulations. [file 1741-7007-12-48-S4.pdf]
