# Supplementary material for: Strong oviposition preference for Bt over non-Bt maize in Spodoptera frugiperda and its implications for the evolution of resistance
Source: BMC Biol. 2014 Jun 16;12:48. doi: 10.1186/1741-7007-12-48 (PMC4094916; doi:10.1186/1741-7007-12-48)

Figure S4. The impact of population dynamics and biased oviposition on the evolution of resistance in computer simulations when resistance to Bt toxins is under partially dominant ($h = 0.1$, **A, B**) and fully recessive ($h = 0$) resistance (**C,D)**. Here we explored the relationship between the density dependent action threshold for spraying refugia (in egg masses per plant) and refuge size under random and damage avoiding oviposition . As in previous models larger refugia had a limited impact on the evolution of resistance when females were avoiding damaged plants. This result was relatively insensitive to spray thresholds, except when refugia were very large. Note that sprays are rarely applied with spray thresholds of 0.8 or above. Once again, small refugia could lead to the simulated populations becoming sinks, which also prevented the evolution of fully recessive resistance. Fecundity was set at 300 eggs per female in all models.

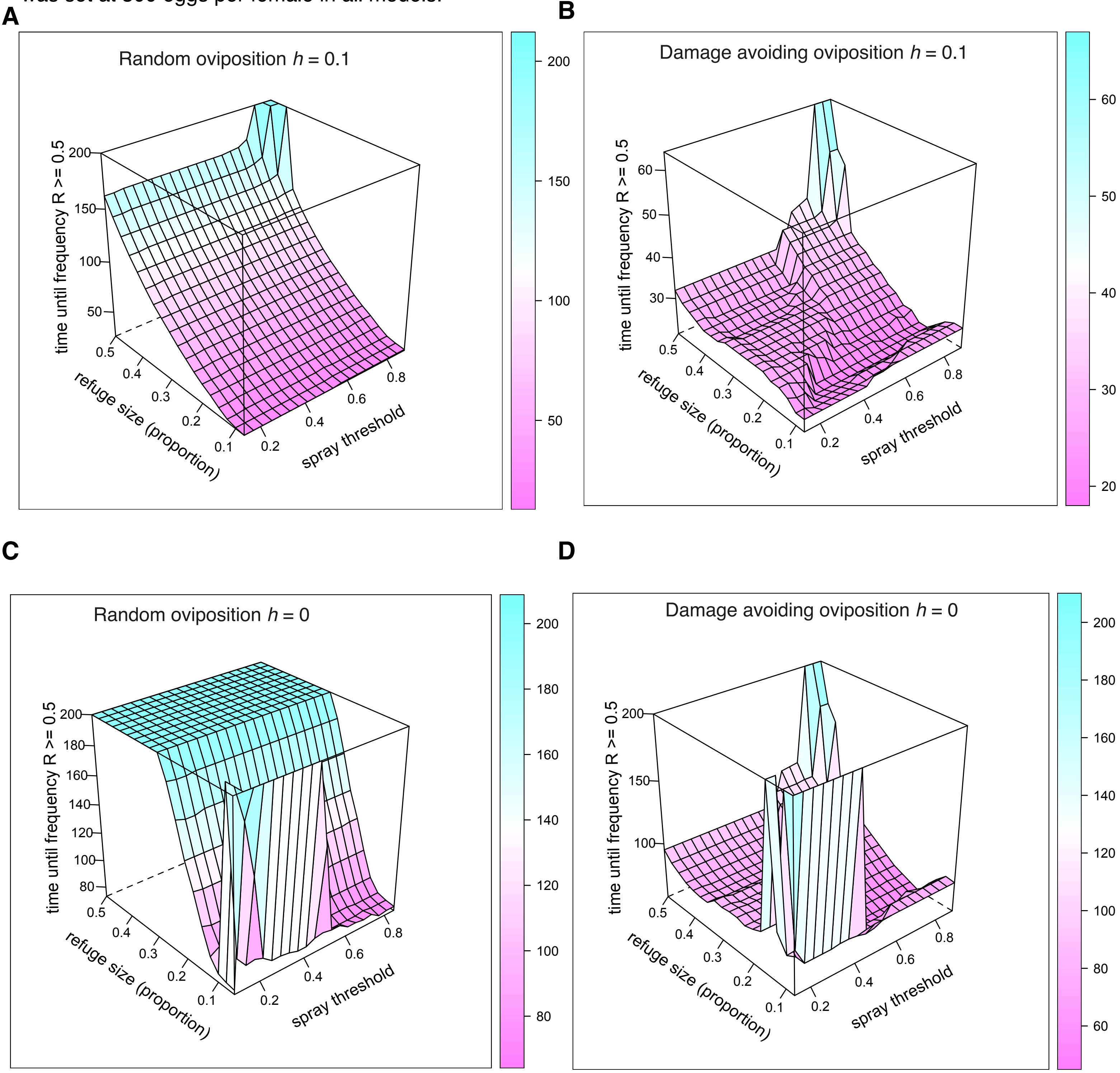

Supplement: Additional file 5: Figure S4 — The impact of population dynamics and biased oviposition on the evolution of resistance in computer simulations when resistance to Bt toxins is under partially dominant (h = 0.1, A, B) and fully recessive (h = 0) resistance (C,D). Here, we explored the relationship between the density dependent action threshold for spraying refugia (in egg masses per plant) and refuge size under random and damage-avoiding oviposition . As in previous models, larger refugia had a limited impact on the evolution of resistance when females were avoiding damaged plants. This result was relatively insensitive to spray thresholds, except when refugia were very large. Note that sprays are rarely applied with spray thresholds of 0.8 or above. Once again, small refugia could lead to the simulated populations becoming sinks, which also prevented the evolution of fully recessive resistance. Fecundity was set at 300 eggs per female in all models. [file 1741-7007-12-48-S5.pdf]
